# The physiology of survival: Heat

**DOI:** 10.1113/EP093192

**Published:** 2025-10-25

**Authors:** Josh Foster, Robert D. Meade, Tom Matthews

**Affiliations:** ^1^ Centre for Human and Applied Physiological Sciences, Faculty of Life Sciences and Medicine King's College London London UK; ^2^ Department of Epidemiology, T.H. Chan School of Public Health Harvard University Boston Massachusetts USA; ^3^ South Asia Adaptation Cluster, Salata Institute for Climate and Sustainability Harvard University Cambridge Massachusetts USA; ^4^ Department of Geography King's College London London UK

## 1 BACKGROUND

Excessive heat is a major health hazard, contributing to ∼1% of all deaths in recent decades (Zhao et al., 2021) and claiming over 250,000 lives in just the deadliest 10 events since 2000 (Matthews et al., 2025). These impacts are also on track to escalate considerably. Warming and moistening of the atmosphere (Blunden et al., 2025; Matthews et al., 2025) have driven an increase in the frequency and intensity of extreme heat events (Perkins‐Kirkpatrick and Lewis, 2020; Raymond et al., 2020), and this is projected to continue with human forcing of the climate system. Compared to recent decades, around 350 million more people in the world's largest cities could be exposed regularly to dangerous levels of heat should global temperature rise to just 1.5°C above preindustrial levels by mid‐century (Matthews et al., 2017). For a more extreme (but plausible) 4°C warming scenario, as many as three billion people by 2100 could encounter uncompensable heat – temperature and humidity combinations so extreme that they would lead to inevitable and dangerous increases in core temperature (Vecellio et al., 2023). The precipitous increases in future heat risk reflect the unfortunate coincidence that the areas of highest risk of heat hazards and fastest population growth coincide, with the tropics and sub‐tropics generally projected to see the largest increases in both (Matthews, 2018).

Even in conditions where body temperature *can* be regulated within homeostatic limits by physiological adjustments, heat can still present as a deadly environmental hazard across the life course. Heat exposure in utero is associated with higher risks of stillbirth, low birth weight and congenital abnormalities (Lakhoo et al., 2025). Infants and very young children have limited behavioural heat avoidance and less efficient autonomic control of body temperature, such that they rely on caregivers for heat avoidance and adequate hydration. Healthy young adults can develop exertional heat illness during physical work in the heat, including forced labour in hot regions or in highly motivated individuals during military exercises (Leon and Bouchama, 2015; Lim, 2018). Older adults are especially susceptible to extreme heat, and more so if they have cardiovascular, renal, respiratory or neurocognitive diseases, or are taking medicines that impair thermoregulation or cardiovascular responsiveness (i.e., β‐blockers) (Fouillet et al., 2006; Layton et al., 2020). The sharp increase in suicide rates and mental health‐related hospital admissions during extreme heat events appears to be age independent (Kim et al., 2019), but more work is needed in this area to better define the most at‐risk groups and underlying pathophysiology. Even without further global warming, maximum air temperature could reach 50°C (over 10°C higher than historical peaks) across major cities in Western Europe, including London and Paris (Noyelle et al., 2023). Thermal physiology is therefore more relevant than ever, as it provides an urgently needed framework to understand the potential impacts of heat beyond precedent, and to help optimise public health efforts to reduce risks.

## 2 THE PHYSICS OF HEAT STRESS

In discussing the health risks of a warming climate, it is critical to define what ‘lethal’ heat is and how it arises. Heat is not inherently lethal and may in fact be used medicinally in safe doses to improve cardiovascular health (i.e., sauna bathing) (Brunt and Minson, 2021). To better understand why heat can *become* lethal, it is necessary to emphasise what the goal of the thermoregulatory system is and how its physiological architecture helps achieve it. Contrary to popular belief and what is still described in some mainstream physiology texts, the thermoregulatory system does not aim to maintain a predefined set point body core temperature (‘set point’ and ‘core’ being the operative words). Instead, the goal of the thermoregulatory system is to maintain an equilibrium state where *thermal energy gain* matches *thermal energy loss* in order to maintain body temperature within safe limits. The concept of a predefined set‐point (typically ∼37°C) is incompatible with the goal of thermal equilibrium, and perceived core temperature regulation is likely a morphological illusion (Mitchell et al., 2025).

The heat balance equation illustrates the physical heat transfer pathways between the body and the environment:

(1) $$S=H_{\mathrm{prod}}\;\pm K\pm C\pm R\pm R_{\mathrm{esp},C}\pm R_{\mathrm{esp},E}\;\pm E\;\left[\mathrm{watts}\;m^{-2}\right]\;$$

where each component is expressed in watts per square metre of skin surface area. *S* is heat storage; *H*_prod_ is heat production from metabolism; the remaining parts relate to heat transfer through convection (*C*), radiation (*R*), conduction (*K*), respiratory convection (*R*_esp,_ *_C_*), respiratory evaporation (*R*_esp,_ *_E_*) and skin surface evaporation (*E*). *H*_prod_ is a source of heat gain, whilst *E* components are a source of heat loss. *K*, *C* and *R* can be a source of heat gain or loss depending on the environmental conditions, clothing, and skin temperature. The heat balance equation (Equation 1) can be rearranged to estimate the *required evaporative heat loss* for heat balance (*E*_req_). Below, we use an example of a hot outdoor work environment (40°C air temp, fully shaded, low wind speed) with moderate clothing insulation (1 Clo), and a heavy work rate (250 W m^−2^). For this example, we assumed a mean skin temperature of 35°C.

(2) $$E_{\mathrm{req}}=250\;\left(H_{\mathrm{prod}}\right)+23\;\left(C\right)+1\;\left(R_{\mathrm{esp},C}\right)-5\;\;\left(R_{\mathrm{esp},E}\right)=269\;W\;m^{-2}$$

Here, the terms *K* and *R* have been removed as they are assumed to be negligible in this scenario. To maintain heat balance, the body, once reaching a steady state, must lose heat through evaporation at a rate of 269 W m^−2^. Sweat evaporation accounts for most of this heat loss, since respiratory evaporation is typically marginal by comparison. The amount of *sweat production* necessary to meet the demands of *E*_req_ depends on the clothing, humidity and wind speed, since these factors modify the sweating efficiency, that is, the ratio of sweat drippage (no heat loss) to evaporation (heat loss at a rate of 2400 J g^−1^ of sweat evaporated). The *signal* that triggers the brain to activate eccrine sweat glands is an *increase* in body temperature. Although a subtle point, it is not well understood (even amongst thermal physiologists) that we *require* an increase in body temperature to activate the afferent fibres that drive heat loss responses (Romanovsky, 2007). Indeed, the 2021 Nobel Prize was awarded for the first description of thermoreceptors and how their activation depends on the temperature they differentially respond to (Earley et al., 2022). Such a view is consistent with thousands of empirical studies where body temperature *stabilises above resting* once thermal equilibrium is achieved (Meade et al., 2024a), where the operating point in body temperature depends on the *required* level of skin blood flow and sweat production (Meade et al., 2024b). If, by contrast, heat loss continued until body temperature reached a predefined set point, body temperature would return to basal values, sweating would cease, heat gain would increase, and body temperature would rise again. In this way, thermoregulatory responses during heat stress do not act to ‘cool’ the body – they act to arrest the upward displacement in body temperature.

In uncompensable scenarios, the maximal rate of heat loss achievable is insufficient to offset heat gain and body temperature cannot stabilise within safe physiological limits, causing extreme physiological stress, which would ultimately become fatal if unattended. These conditions are well understood and the source of fervent scientific research (Leon and Bouchama, 2015). However, a less considered concept is that the vast majority of thermal injuries, from cardiovascular events to mental health outcomes, likely occur as a consequence of normal thermoregulation, not a failure of it (Meade et al., 2024b). Even in compensable zones where body temperature stabilises, thermoregulation can stress multiple organ systems. Consequently, the human body has multiple possible failure points when dealing with elevations in thermal energy gain. Here, we will discuss some common failure points across multiple populations, with the exception that psychiatric disorders are largely unknown in their underlying pathophysiology related to heat due to the involvement of multiple indirect effects (i.e., poor sleep quality, medications, etc.).

## 3 PHYSIOLOGICAL MECHANISMS UNDERLYING HEAT‐RELATED HEALTH RISKS

Long before reaching high levels of thermal strain (e.g., those associated with thermoregulatory failure), cardiovascular adjustments would have already arisen (Crandall and Wilson, 2011Crandall *et al.*, 2011). It is in these cardiovascular adjustments that common pathways mediating many heat‐related health problems can be found. Early into any heat stress exposure, the initial rise in skin temperature triggers reflex cutaneous vasodilation to increase whole body skin blood flow (Rowell et al., 1969). This increase in skin blood flow is critical to (1) move heat from the core to the skin, (2) minimise *dry* heat gain in exceptionally hot environments, and (3) increase water vapour pressure over the skin, improving sweat evaporative efficiency. Importantly, the cardiovascular adjustments to heating underpin a number of possible adverse health effects. First, widespread cutaneous vasodilation triggers a reflex increase in cardiac output to maintain mean arterial pressure (MAP) (Rowell et al., 1969). As body temperature rises, so does vasodilation, increasing the required cardiac output to maintain MAP. In individuals with heart failure or on β‐blockers, it is possible that during extreme heat stress, cardiac output may not be augmented sufficiently to maintain MAP, resulting in cardiovascular decompensation (Foster et al., 2025; Meade et al., 2025). Age over 65 years and certain cardiovascular diseases impair reflex cutaneous vasodilation in response to heating (Greaney et al., 2015; Stanhewicz et al., 2016). Whilst less vasodilation means less requirement to augment cardiac output for the maintenance of blood pressure, compromised thermoregulation invariably results in a greater body temperature required to achieve thermal balance (McKenna et al., 2023).

Second, non‐cutaneous vasoconstriction, particularly in the renal and splanchnic vascular beds, helps maintain MAP and reduces the burden on the heart to augment cardiac output (Hall et al., 1999; Minson et al., 1998, 1999). Whilst beneficial for thermoregulation, the relative ischaemia to central organs can directly cause tissue damage, evidenced through either an increase in gastrointestinal permeability or markers of acute kidney injury in healthy humans exposed to extreme heat (Chapman et al., 2020; Lambert, 2004; McKenna et al., 2025b; Selkirk et al., 2008). Although the mechanistic pathways are poorly described in humans, animal models suggest that tissue injury is likely caused by a combination of hypoperfusion‐induced ATP depletion and acidosis, and reactive oxygen species‐mediated damage during reperfusion (Hall et al., 1999, 2001). Severe or prolonged disruption of the gastrointestinal barrier can lead to microbial translocation from the gut to systemic circulation, potentially leading to disseminated intravascular coagulation, as was shown in older people admitted to the intensive care unit during the 2003 heat wave in France (Huisse et al., 2008). Susceptibility to heat‐induced kidney failure likely depends on whether individuals have underlying renal disease. However, during occupational heat stress with daily heat and dehydration, evidence for chronic kidney injury is evident in otherwise healthy individuals (Flouris et al., 2018).

Central redistribution of blood flow to the periphery may account for the adverse birth outcomes linked to excessive maternal heat exposure (Chersich et al., 2020). Whilst maternal thermoregulation has been shown to be unimpaired (at least in healthy pregnancy) relative to non‐pregnant women (Ravanelli et al., 2019), fetal responses are poorly understood. Simply relying on maternal thermoregulation is a poor indicator of fetal health since fetal temperature may be up to 1°C higher than maternal measurements (Laburn et al., 2002; Lavesson et al., 2018). In an uncomplicated pregnancy, short but intense heat exposure increases fetal heart rate without compromising fetal blood flow (Vähä‐Eskeli et al., 1991). However, in hypertensive pregnancies, acute heat stress increased uterine vascular resistance, suggesting a reduction in fetal blood flow (Pirhonen et al., 1994). More recently, Bonnel *et al.* demonstrated an average 22 beats min^−1^ increase in fetal heart rate whilst monitoring pregnant subsistence farmers in The Gambia (Bonell et al., 2022). Of the 91 participants, only 53% went on to have a ‘normal’ birth outcome, with the remainder being small for gestational age (26%), preterm (13%), low birthweight (13%) or stillbirth (3%). Here, 22/40 (55%) participants showed increases in umbilical artery resistance. Thus, it appears that the level of heat strain is positively related to fetal health risks, with core temperature appearing to be a poor predictor of these responses. Little is known about how repeated heat exposure throughout gestation amplifies risks; however, it is likely that heat exposure in critical early development periods (i.e., first trimester) carries the greatest risk.

It is not possible to discuss in detail all the pathways of heat‐induced pathophysiology in all vulnerable groups. In some cases, our physiological understanding is simply too coarse to provide such an insight, particularly related to the exacerbation of respiratory and psychiatric conditions with heat exposure. Instead, we provide these examples simply to highlight that heat can become a major health hazard without the necessity of a critically high core temperature or exposure to fully uncompensable environments. Heat susceptibility is primarily driven by highly conserved and fast‐acting physiological responses that treat the thermal homeostasis of the host as a high‐priority variable, often at the cost of compromised blood flow to or stress on implicated organ systems. A full physiological understanding of the factors that modify heat‐related health risks in diverse populations is urgently required to build accurate thermophysiological models, which can predict risk due not only to failure of thermoregulation, but also to the sustained strain on the body's other physiological systems that thermoregulation incurs. Such models will also need to be highly individualised. Numerous inter‐ and intra‐individual factors are well known to modify physiological response to heat exposure, including hypohydration, medications, hypoxaemia, acclimatisation, physiological status and underlying disease. Currently, however, limited detail is available on how these mechanisms interact with each other to modulate heat‐related health risks, especially over the increasingly long‐duration heat exposures being experienced around the globe.

## 4 HIERARCHY OF COOLING APPROACHES

Whilst the pathophysiological mechanisms underlying heat illness are dynamic and poorly understood, the first common pathway involved is heat gain itself (*S* in Equation 1). Adequately removing heat sources or significantly improving the extraction of heat from the body can prevent heat‐related health issues from occurring. For any cooling strategy to be deemed effective, it must reduce the thermal energy absorbed and stored by the body, resulting in a lower body temperature and therefore less activation of auxiliary responses (e.g., cardiovascular adjustments), at heat balance. Electric fans are a good example of this conceptual problem. Using a fan when air temperature > skin temperature means that additional air flow increases *dry* heat gain, akin to how a convective oven or air fryer works (Morris et al., 2019a). However, there are nuances where a fan *may* still provide a small net cooling effect by increasing sweating efficiency, and thus *E*, evaporative heat loss. The impact can be meaningful in exceptionally hot and humid environments (Foster et al., 2022), but such environments are very rare and realistically will require more aggressive cooling measures to ensure the safety of the occupants/workers. Importantly, in most extreme heat situations, the effects are simply too small and nuanced to declare fans are an effective solution when air temperature is >∼35°C, especially as they may elevate heat energy gain, which is harmful in hot climates (Foster et al., 2022; Meade et al., 2024c; Morris et al., 2019a).

Electric fan use is one example, but the problem extends to other modalities typically studied in the physiology literature, such as limb immersion in cold water, skin wetting and ice slurry ingestion. Additionally, built environment modifications may result in small reductions in air temperature, especially where designs currently elevate heat above background levels (Wilby et al., 2021), but the question remains as to whether such modifications are powerful enough to decrease the risk of heat‐related adverse health outcomes. For a solution, or combination of solutions, to be universally effective from a physiological point of view, it should result in a sustained and powerful reduction in whole body heat storage (*S*, Equation 1). Such a reduction would meaningfully decrease the required body temperature elevation needed for heat balance and associated physiological strain. The only solution that achieves this goal is reducing exposure to the heat, either through relocation to a cooler space or by actively cooling the environment using air conditioning (ideally powered by renewable energy sources) or some other powerful ambient cooling approach (e.g., heat pumps or swamp coolers, but these have their own limitations). Consequently, such approaches should currently be considered as the first line of defence for widespread protection of heat‐vulnerable people.

Total removal from the heat will reduce *S* to near zero, and therefore the required body temperature to achieve heat balance (Meade et al., 2023). However, removal from the heat is often not an option, particularly in hospitals, care homes and certain occupational scenarios. In such conditions, a combination of personal approaches should be considered, based on their effectiveness and feasibility. Water has ∼20 times the thermal conductivity of air, such that whole body or limb immersion can potentially extract a significant amount of heat from the body. The magnitude of heat extraction depends on the surface area of skin exposed, the temperature of the water and motion (still water provides less cooling). Thus, whole‐body cold‐water immersion is the most effective method to extract heat from a hot body quickly, such that it is considered the gold standard approach for treating excessively high core temperatures and preventing heat stroke (Casa et al., 2007). In the home or at work, overt whole body cold water immersion ∼10°C is uncommon due to the stressful cold shock response it can invoke (Tipton, 2018). However, water temperature merely needs to be lower than body temperature, such that water temperature of 20°C is more tolerable but can still extract significant heat from a hot person (Proulx et al., 2003). If no environmental modifications are made, core temperature will rebound back to the pre‐cooling value, providing ∼1–2 h of benefit depending on the environmental heat severity. If water is available, limb immersion or dousing the skin has also been shown to reduce thermal and cardiovascular stress, albeit with a deep body temperature remaining well above resting values (McKenna et al., 2025a; Morris et al., 2019b). However, whilst physiological studies provide promising evidence of the effectiveness of such strategies, considerable work remains in translating these findings to real‐world environments. For example, prolonged water‐based cooling may not be feasible for some groups, such as older adults with diabetes, because of elevated risks of skin maceration, infections, or pressure ulcers.

## 5 CONCLUSION AND FUTURE DIRECTIONS

The physiology of survival in the heat makes clear that the critical determinant of safety is the ability to reduce or prevent excessive heat storage (*S*). If *S* remains positive, body temperature will rise, physiological strain will escalate and vulnerable organs or tissues will eventually fail. Cumulative exposure to even modest body temperature rises may predict a range of adverse effects, ranging from heart attacks and kidney injuries to psychiatric episodes leading to psychosis or suicide. Although we have a detailed understanding of how healthy humans maintain heat balance under controlled laboratory conditions, much less is known about how repeated, cumulative and interacting stressors play out in diverse populations and real‐world settings. The challenge is not simply to prevent uncompensable extremes, but to recognise that most heat‐related morbidity and mortality likely occur in compensable heat stress, not due to a failure of thermoregulation, but because of it.

What is urgently needed now is a bridging of approaches. Epidemiological studies provide scale, but they cannot extrapolate safely to unprecedented future climates. Physiological models offer mechanistic insight but require more attention to interacting factors such as hydration, medications, or disease, and few, if any, available models consider the continuum of physiological responses to heat and how they contribute to risk. Engineering and behavioural strategies provide practical solutions, but their effectiveness must be quantified in terms of whole‐body heat balance and associated physiological strain. In extreme heat, no single cooling measure, be it a fan, water ingestion, or insulation retrofit, will likely suffice in isolation. To meaningfully protect populations, we must prioritise integrated approaches that demonstrably drive *S* to manageable levels. Identifying what those levels are, and how we maintain them in practice is a critical goal for thermophysiological research.

## AUTHOR CONTRIBUTIONS

All authors have read and approved the final version of this manuscript and agree to be accountable for all aspects of the work in ensuring that questions related to the accuracy or integrity of any part of the work are appropriately investigated and resolved. All persons designated as authors qualify for authorship, and all those who qualify for authorship are listed.

## CONFLICT OF INTEREST

The authors declare they have no conflicts of interest.

## FUNDING INFORMATION

No external funding contributed to the formulation of this article.
